# Stratospheric controlled perturbation experiment: a small-scale experiment to improve understanding of the risks of solar geoengineering

**DOI:** 10.1098/rsta.2014.0059

**Published:** 2014-12-28

**Authors:** John A. Dykema, David W. Keith, James G. Anderson, Debra Weisenstein

**Affiliations:** 1 School of Engineering and Applied Sciences, Harvard University, One Brattle Square, Cambridge, MA 02138, USA; 2 Department of Chemistry and Chemical Biology, Harvard University, Mallinckrodt Link Building, 12 Oxford Street, Cambridge, MA 02138, USA; 3 Harvard Kennedy School and School of Engineering and Applied Science, Pierce Hall, 29 Oxford Street, Cambridge, MA 02138, USA

**Keywords:** geoengineering, solar radiation management, stratosphere, balloon, ozone depletion

## Abstract

Although solar radiation management (SRM) through stratospheric aerosol methods has the potential to mitigate impacts of climate change, our current knowledge of stratospheric processes suggests that these methods may entail significant risks. In addition to the risks associated with current knowledge, the possibility of ‘unknown unknowns’ exists that could significantly alter the risk assessment relative to our current understanding. While laboratory experimentation can improve the current state of knowledge and atmospheric models can assess large-scale climate response, they cannot capture possible unknown chemistry or represent the full range of interactive atmospheric chemical physics. Small-scale, *in situ* experimentation under well-regulated circumstances can begin to remove some of these uncertainties. This experiment—provisionally titled the stratospheric controlled perturbation experiment—is under development and will only proceed with transparent and predominantly governmental funding and independent risk assessment. We describe the scientific and technical foundation for performing, under external oversight, small-scale experiments to quantify the risks posed by SRM to activation of halogen species and subsequent erosion of stratospheric ozone. The paper's scope includes selection of the measurement platform, relevant aspects of stratospheric meteorology, operational considerations and instrument design and engineering.

## 1. Scientific perspective

Solar radiation management (SRM) supposes that deliberate addition of aerosol to the stratosphere could reduce climate risks by partially offsetting the radiative forcing from accumulating greenhouse gases. The past few years have seen a tremendous surge in research examining the efficacy and risks of SRM. A large body of research has used general circulation models (GCMs) to examine the climate response to SRM forcing. Most of these have used very simple models of stratospheric aerosol. For example, many simply adjust the top of the atmosphere radiative forcing [1–3]. A more limited set of studies have used interactive aerosol models in GCMs, but, in most such studies, to date, the aerosol size distribution has been prescribed [4–6], and changes in climate (temperature, precipitation) predicted without chemical feedbacks.

There have been studies using two-dimensional models with aerosol dynamics in which the size distribution is allowed to freely evolve [7,8], but these two-dimensional models have important limitations. For example, they cannot accurately treat stratosphere–troposphere exchange nor can they examine zonal heterogeneity. A few models have employed aerosol dynamics within a three-dimensional framework [9,10] but without any chemical interactions with ozone. All such studies find that aerosol particle distributions in a geoengineered stratosphere could be larger than observed after the 1991 Mt. Pinatubo eruption, and that the size distribution is sensitive to the injection method, location and frequency.

While a set of studies have examined the impacts of SRM on ozone chemistry, all of the studies have used simple prescriptions of aerosol distributions [6,11] or aerosol distributions calculated in off-line models [7]. This is a serious limitation as the distribution of aerosol surface area can have a profound effect on ozone chemistry, with feedback effects also linking ozone chemistry to temperature and dynamics. Tilmes *et al.* [12] found that geoengineering could greatly enhance chlorine activation in the polar regions during cold winters, possibly enlarging the region of polar ozone depletion. The Heckendorn *et al.* [7] study using a chemistry-climate model found that aerosol heating near the tropical tropopause induced by geoengineering modified stratospheric water vapour, which resulted in additional ozone depletion.

To first order, between the local tropopause and approximately 30 km altitude at mid-latitudes, ozone concentrations are controlled by a combination of transport and photochemical production and loss, with photochemical control increasing with increasing altitude. At altitudes above approximately 30 km in summer, ozone concentrations are dominantly controlled by catalytic photochemistry. Therefore, the assessment of SRM depends on the coupling of chemistry and dynamics in the lower stratosphere. Furthermore, it has been demonstrated that the catalytic chemistry is highly sensitive not only to aerosol surface area density (SAD), but also to water vapour [13]. Elevated levels of lower stratospheric water vapour constitute an additional uncertainty and risk factor for ozone and SRM.

### (a) Catalytic chemistry

In 1994, it was demonstrated by direct *in situ* observations of the rate-limiting radicals by Wennberg *et al.* [14] that chemical ozone loss in the lower stratosphere is dominated by catalytic removal through reactions with the hydrogen–oxygen (HO_*x*_) radicals OH and HO_2_. This represented a major turning point in our understanding of ozone loss from the previously held view that the catalytic loss of ozone was rate limited by NO_*x*_ radicals, specifically NO and NO_2_ in the lower stratosphere. In fact, because HO_*x*_ radicals are the dominant rate-limiting radicals in this system, and because reactions with NO_*x*_ radicals are the dominant reactive pathways converting the rate-limiting HO_*x*_, ClO_*x*_ (ClO and Cl) and BrOx (BrO and Br) radicals to their non-catalytic inorganic forms, the NO_*x*_ radicals become the buffering species rather than the catalytic species in ozone removal. As a result, with a decreasing concentration of NO_*x*_ species, the rate of ozone catalytic loss in the lower stratosphere increases, because the rate-limiting radicals HO_2_, ClO and BrO that are removed by NO_*x*_ increase in concentration.

Large ozone losses that occur over the polar regions result directly from heterogeneous reactions involving inorganic chlorine [15]. These reactions serve primarily to transform inorganic chlorine (principally HCl and ClONO_2_ that constitute approx. 97% of available inorganic chlorine) into the rapidly photolysed intermediates Cl_2_ and HOCl, followed by reaction of the product Cl atoms with ozone to form the primary catalytically active chlorine radical, ClO. What proved to be of particular importance from the NASA SAGE III ozone loss and validation experiment mission [16–20] was that examination of conditions in the Arctic lower stratosphere coupled with emerging results from laboratory experiments showed that the dominant pathway for chlorine activation appears to be on simple, ubiquitous, cold sulfate–water aerosols [15,21–24]. Thus, it is both temperature and water vapour concentration in combination with simple binary sulfate–water aerosols that primarily determine the kinetics for rapid chlorine activation.

Enhanced ClO that results from increases in sulfate aerosols or water vapour in the stratosphere [17,18] can accelerate ozone destruction primarily through one of two catalytic reaction cycles: the ClO dimer mechanism, or a coupled bromine and chlorine mechanism [25]:

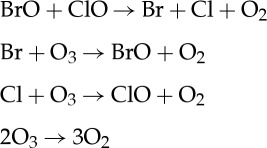



Even small changes in the lower stratosphere can have significant consequences for ozone, as the heterogeneous reactions that set the threshold conditions for chlorine activation are extremely sensitive to temperature, water vapour and reaction aerosol surface area. We know from the injection of sulfates following the volcanic eruption of Mt. Pinatubo [26] that the impact on ozone of enhanced sulfates can be significant.

Accurate photochemical models for the lower stratosphere are necessary to quantitatively assess changes to ozone loss rates resulting from increased stratospheric aerosol loading. Currently, there are significant uncertainties in the rates of key reactions necessary to forecast ozone loss and recovery. Monte Carlo scenario simulations of the impact of the known uncertainties in these kinetic parameters identify chlorine and bromine reactions as the dominant driver of uncertainty in ozone loss rates [27]. Further uncertainty in future ozone loss rates is driven by uncertainty about the meteorological conditions under which these reactions will take place.

### (b) Water vapour and dynamics in the lower stratosphere

**Figure 1.**
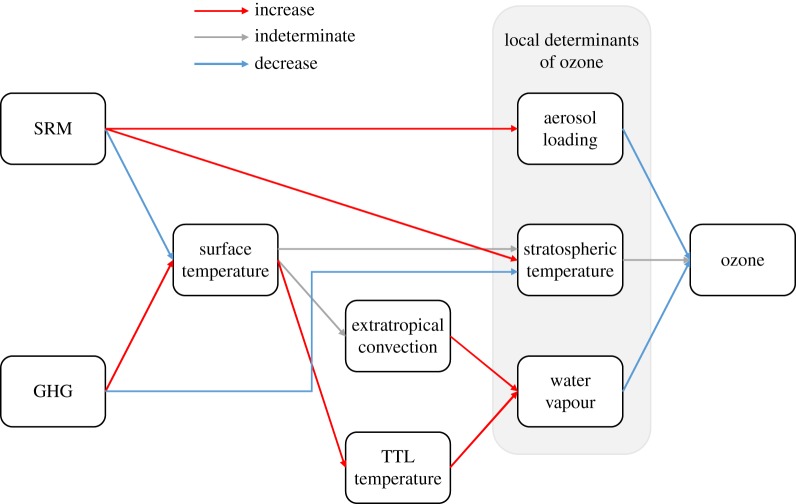
Schematic of interactions between green house gas (GHG)-driven climate change, SRM and stratospheric ozone. A red arrow denotes an interaction where an increase in the quantity on the left generally causes an increase in the quantity on the right; a blue arrow denotes the converse; and a grey arrow is used for indeterminate cases. Sulfate aerosol causes direct radiative heating of the lower stratosphere and perhaps of the tropical tropopause layer (TTL). SRM would introduce a net negative radiative forcing that would offset some impacts of the positive forcing from increased GHGs. The combined effects of increased surface aerosol density, stratospheric temperature decreases and water vapour increases could substantially increase photochemical ozone losses. Conversely, SRM aerosol might decrease stratospheric water vapour, an offsetting effect. The purpose of SCoPEx is to reduce the uncertainty in our knowledge of relevant aerosol processes and this photochemistry through *in situ* perturbation experiments.

Changes in stratospheric water vapour content play a central role in mediating the stratosphere's response to greenhouse gas-driven climate change and to the use of SRM to offset such changes. A combination of radiative [28–32], dynamical [33,34] and chemical processes [35] associated with water vapour complicate the prediction of ozone loss rates in a deliberately engineered climate (figure 1). We first describe the relevant determinants of water vapour in the current climate, and then speculate about the interaction of climate change and SRM.

Observations of stratospheric water vapour indicate a mixed pattern of increases and decreases over decadal time scales [36–38]. Projections based on coupled chemistry–climate GCMs suggest a secular increase in stratospheric water vapour over 50 years [39]. Increased stratospheric water vapour concentrations will add to the radiative forcing of climate and tend to exacerbate ozone loss. Other than CH_4_ oxidation, H_2_O enters the stratosphere either by transport into the stratosphere through the tropical tropopause or by dynamical mixing of tropospheric air into the lowermost stratosphere in mid-latitudes. Dessler *et al.* [40] have demonstrated a robust correlation between increased surface temperatures and increased stratospheric H_2_O, but we lack a high-quality mechanistic understanding of either pathway.

Recent findings have drawn attention to an unexpected source of tropospheric water vapour to the stratosphere. *In situ* measurements of water vapour in the lower stratosphere show a significant frequency of elevated values, occurring in approximately 50% of summertime flight observations over the USA [35]. The convective origin of these water vapour measurements is established by simultaneous *in situ* observations of H_2_O and the HDO isotopologue [41,42], differentiating between direct convective injection- and other temperature-controlled pathways linking the troposphere and stratosphere [41,43,44]. Convective injection of water vapour as reported in [35] can occur in storm systems that are approximately 50 km across, with smaller domains of high-altitude injection embedded within them [45,46]. The elevated concentrations of water can spread to 100 km or more in horizontal extent within a few days, and may remain at the elevated levels over a period of days.

The existence of these regions of substantially enhanced water vapour may represent an important pathway for water vapour entry into the lower stratosphere as surface temperatures warm. A coherent understanding has yet to coalesce unifying all observational and theoretical lines of evidence [47]. Recent work by Ploeger *et al.* [34] and by Homeyer *et al.* [48–50] have brought emphasis to the competition between (i) horizontal water vapour transport in the lower stratosphere from subtropics to high latitudes and (ii) deep stratospheric convective injection of water vapour over the USA in summer, respectively.

Now we consider the impact of an increased sulfate aerosol loading in the lower stratosphere. First, there will be a direct impact on ozone concentration through the halogen activation pathways described above [11]. Second, there will be competing indirect effects that have received little attention to date. On the one hand, SRM sulfates may decrease stratospheric water vapour by decreasing tropical tropopause temperatures [50] or by decreasing the energy that drives subtropical convective injection. On the other hand, an increase in sulfate aerosol loading in the tropical tropopause layer will increase radiative heating rates and so raise temperatures, potentially increasing stratospheric water vapour concentration. The increased stratospheric water vapour could produce a wetter stratosphere, leading to much faster ozone losses. Conversely, the net effect of SRM could be less ozone loss if the induced cooling reduces transport of water vapour into the lower stratosphere. Figure 1 illustrates these competing pathways.

Taken together, these considerations speak to the need to improve understanding of (i) the radiative impact of SRM aerosols, (ii) the potential for enhanced ozone loss under conditions of high water vapour, and (iii) the processes that determine the transport of water vapour into the lower stratosphere.

### (c) The necessity for direct experimentation in the lower stratosphere

The stratospheric controlled perturbation experiment (SCoPEx) aims to advance understanding of the risks and efficacy of SRM. No single scientific effort stands alone. Laboratory experiments, for example, play an essential role in understanding stratospheric processes. Sophisticated chemical reactors have been developed to simulate stratospheric conditions and provide controlled environments to observe reactions of free radicals [51–53]. Particle chambers have been built to study the dynamics of aerosol particles under controlled environmental conditions. Laboratory investigations cannot, however, simultaneously meet all conditions necessary to quantify uncertainties associated with physical processes in the stratosphere. Laboratory systems, for example, are limited in their ability to realize gas flows that do not interact with the chamber walls, and interactions with the walls interfere both with chemical kinetics and with the dynamics of particles. Nor can laboratory experiments quantitatively simulate the catalytic role of UV photons on gas- and liquid-phase constituents with the correct solar spectrum and a realistic population of reactive intermediates.

The consequences of the stratosphere's multi-scale variability are hard to predict, particularly in the case of heterogeneous reactions on aerosols, which are known to have strong nonlinear dependencies on temperature. This unpredictability is increased by the uncertain knowledge of the inventory of radical reservoir species and aerosol types and microphysics. Experiments executed *in situ* in the lower atmosphere are therefore a necessary complement to laboratory experiments if we are to reliably and comprehensively quantify the reactions and dynamics defining the risks and efficacy of SRM.

Aircraft experiments revolutionized stratospheric science by exploiting the natural variability of the stratosphere's chemical composition by examining how one quantity covaries with another, e.g. ClO with O_3_ in the polar regions [54]. These ‘partial derivative’ experiments benefit from long flight tracks that allow us to accumulate robust statistics as a wide range of variability is observed. Experiments to understand the risks and efficacy of SRM will sometimes be able to use the same strategy when natural variability covers the relevant parameter space. Perturbative experiments allow us to extend scientific investigations to look outside the natural range of variability and to better control independent variables.

Moreover, it is plausible that conclusions reached with direct, *in situ* observations within the lower stratosphere itself will greatly simplify the scientific arguments, providing a better basis for public discussion and policy-making about the risks of SRM than computer models and laboratory experiments alone.

Another essential need for *in situ* experiments is to determine the size distribution of aerosol particles as a function of time following injection of a sulfur-bearing gas. The size distribution will depend on the rate at which H_2_SO_4_ gas nucleates into particles; the size and number concentration of those particles will determine their coagulation rate into larger particles; and the rate of plume expansion and dilution will determine the time evolution of the size distribution. Dynamical effects within the first milliseconds will determine nucleation properties, whereas the degree of spatial heterogeneity in the plume as it expands will affect the later size distribution of the particles. Smaller mean particle sizes or broader distributions will result in greater sulfate SAD, producing a larger perturbation to stratospheric chemistry and a greater risk of ozone depletion. Larger mean particle sizes would lead to faster sedimentation rates, a shorter stratospheric lifetime for sulfate particles, and less radiative forcing per unit of sulfate [7,8,10].

## 2. Experimental approaches

### (a) General requirements for *in situ* experimentation

The fundamental experimental protocol for SCoPEx consists of first seeding a small volume with sulfate particles or water vapour, either individually or in combination. The chemical evolution as a function of time within the volume must then be measured with sufficient sensitivity to detect the progress of the photochemical reactions that limit the rate of ozone loss in the mid-latitude lower stratosphere. The time evolution of the aerosol size distribution must be measured with adequate resolution to compute the aerosol radiative properties, settling rate and contribution to halogen activation. Requirements for the implementation of this experiment include

— the experimental system must be capable of injecting controlled amounts of water, and sulfate or other aerosol into a defined well-mixed volume in the stratosphere;
— the system must track the seeded volume continuously, so that it can be re-entered at will, and it should monitor the volume's geometry;
— the experimental duration must exceed 24 h, because the ozone chemistry is strongly modulated by the diurnal cycle of UV irradiance;
— disturbance of seeded volume by *in situ* sampling should be minimized;
— for sulfates, the system must produce aerosol with size distributions relevant to tests of SRM deployment (0.1–1.0 μm radii);
— to minimize environmental risk, the amount of injected material should be as small as possible, consistent with given limitations arising from signal-to-noise (SNR) and plume dispersion during the experimental period; and
— the system must sample the seeded region *in situ* to obtain a sequence of observations of the key species ClO, BrO, O_3_, H_2_O, HDO, aerosol number density and size distribution, NO_2_, HCl, temperature and pressure.

Lower stratospheric chemistry experiments were often conducted by balloon in the 1970s and 1980s. More recently, the existence of high-altitude aircraft and sophisticated, compact chemistry payloads has shifted aircraft into the dominant role for these investigations. The optimum platform for undertaking an investigation such as SCoPEx can be determined through consideration of the experimental requirements.

### (b) Defining an optimum experimental platform

A perturbative experiment must take repeated measurements of a small perturbed volume to study its temporal evolution. This requirement points to platforms that have long endurance. The need to monitor the chemistry over more than a single diurnal cycle to observe the solar influence on the photochemistry demands an endurance of greater than 24 h. In order to satisfy the full set of requirements given in §2*a*, the observing system must be able to maintain float altitude for an extended period of time, it must be able to navigate horizontally to (i) perturb the selected volume with injection of sulfate aerosol and/or water, (ii) track the position of and follow the perturbed region (so as not to lose it) as it drifts with slow background horizontal winds, and (iii) repeatedly sample the seeded region without introducing either excessive turbulence or chemical perturbation.

A propelled balloon has significant advantages over aircraft in meeting these requirements. The required endurance is well within the capabilities of super-pressure balloons (SPBs) [55–60]. Monitoring and tracking the perturbed volume is greatly simplified by a measurement system that can drift with ambient winds. No available aircraft meets our combined requirements of endurance and payload capacity. Finally, a balloon can take advantage of the relatively quiescent state of the background stratosphere [61,62] to minimize the size of the perturbed volume required to observe the reactions of interest, thereby reducing environmental risk.

### (c) Creating and monitoring a well-mixed, chemically perturbed volume

The experimental protocol for SCoPEx depends on understanding the dispersion processes that define the geometry and temporal behaviour of the perturbed volume. The unique characteristics of the mid-latitude lower stratosphere are advantageous in simplifying the implementation of perturbation experiments such as SCoPEx. First is the most obvious—the stratosphere is stable against vertical exchange, because the intrinsic temperature increase with altitude severely restricts vertical exchange. Thus, the ‘stratosphere’ designation. Second, over the USA in summer, the lower stratosphere in the altitude range from 18 to 23 km is remarkably quiescent with respect to both zonal flow velocities and shear. During May through September, lower stratospheric temperatures in the region of 50–70 hPa are in the range of 200–214 K, and wind speed is in the range of 2–7 m s^−1^. Turbulent mixing in the background stratosphere is dominated by large regions of minimal turbulent activity, punctuated by small ‘pancakes’ of turbulence [61,62] where energetic mixing occurs. In these regions, a small perturbed volume will mix very slowly with surrounding air. The slow dilution of passive tracers in the stratosphere has been analysed by Newman *et al.* [63] using high-altitude (70–100 hPa) observations of rocket plume dispersion that define the rate of horizontal spreading from a point source.

Molecular diffusion is too slow, and background turbulent mixing too unpredictable to allow the creation of well-defined and well-mixed experimental perturbations. Some external mixing is required to create a well-defined volume where the reactions of interest can occur. We ran numerical simulations to see if this could be achieved by the atmospheric mixing in the wake of a propelled balloon.

Our simulation was driven by background meteorological conditions determined by combining inspection of wind data from reanalysis [64] and radiosonde data with a survey [65–67] of the literature on stratospheric turbulence. Based on these efforts, we defined base and limiting cases with diffusion coefficients of 0.01 and 1.0 m^2^ s^−1^, vertical shears of 0 and 2 m s^−1^ km^−1^, and balloon airspeeds of 1 and 5 m s^−1^, respectively. The simulation assumed a 60 m diameter balloon and a 20 m tether to the suspended payload. The base case diffusion coefficient was chosen as a most representative value on small spatial scales for quiescent stratospheric air based on a review of *in situ* measurements [68]. The propeller and balloon parameters were chosen to approximately represent a range of possible engineering designs rather than one specific finalized design. Aerodyne Research, Inc. (Billerica, MA) provided a computational fluid dynamics (CFD) simulation (G Magoon, J Peck, R Miake-Lye 2013, unpublished work) of the plume covering the initial development of the turbine propeller wake over the first 45 min following injection. This simulation used the OpenFOAM [69] CFD code run in a Reynolds-averaged stress mode modified to represent the dispersion of a passive tracer. We then used our own advection–diffusion code driven by reanalysis winds to examine how the plume might evolve over a 24 h period following release. This code uses a second-order numerical scheme with a fixed diffusion coefficient to compute kinematic parcel trajectories (see Bowman *et al.* [70] for a review of related models).

**Figure 2.**
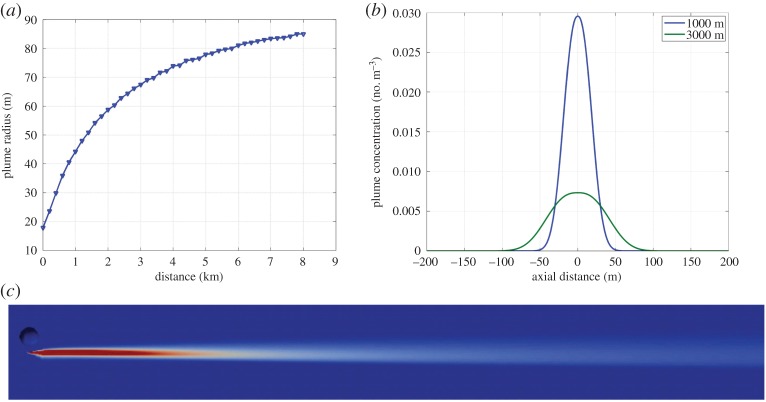
Results of CFD calculations for balloon physical configuration and propulsion assuming 1 m s^−1^ at 20 km altitude. (*a*) The plume radius—defined by a passive tracer concentration of 5×10^−3^ of the initial peak concentration found on the centreline of the well-developed plume—as a function of the distance downstream (km). Plume dispersal will be dominated by wakes generated by balloon motion. The plume initially expands rapidly, slowing after a few hours towards an asymptotic radius. (*b*) The tracer concentration at distances of 1000 and 3000 m downstream as a function of plume radius. (*c*) The concentration of a passive tracer (arbitrary units) released from the balloon gondola as it travels right to left.

The results for the base case (figure 2) show that a well-developed plume forms in the propeller wake with an initial radius of about 20 m. At a distance of 8 km from its initial injection, the plume radius grows to about 85 m (or order 100 m for defining a nominal plume volume). These results suggest that the propeller wake can be used to create a well-mixed area in which to perform the perturbation experiment. The propelled balloon payload in SCoPEx thus performs two interdependent tasks. First, it allows us to create a perturbed region, and, second, it allows us to manoeuvre around that region, so its evolution can be tracked and monitored over time.

The rate of plume dispersion is crucial to (i) forming an appropriately sized particle distribution using the methods of Pierce *et al.* [8], (ii) understanding the distribution of induced chemical perturbations within the plume, and (iii) understanding how the plume evolves in three dimensions to ensure that the payload can re-enter the plume multiple times during the experiment. Note that the experimental design is based on probing the variation of observed chemistry with simultaneously observed perturbations of H_2_O and aerosols. Therefore, while plume modelling is needed for operations, the accuracy of scientific results does not strongly depend on our ability to model concentrations in the plume.

These CFD results show that a correctly designed propeller can provide this mixing, but it results in a small turbulent disturbance relative to a similar experimental approach executed by aircraft. Analysis [71] of aircraft contrails normalized to match results from the Concorde [72] indicates that an aerosol perturbation generated in the wake of a stratospheric plane will grow to approximately 250 m diameter after 2.5 h. This implies that over 30 times as much sulfur would be required relative to a propeller-generated plume (with a radius of order 100 m after 2.5 h), with a proportional increase in physical risk. The relatively rapid growth of the aircraft plume also means that, for each pass back through the plume to make chemical measurements, a significantly larger fraction of the aerosol plume will be disturbed and vigorously mixed with background air. This disturbance of the plume means that the sampling regions must be further apart, meaning a longer plume is required to achieve the same number of samples.

### (d) Physical and chemical measurements

The instrumentation necessary to introduce the chemical perturbation, and to perform chemical and meteorological measurements and plume tracking (table 1) is chosen according to the following rationale:

**Table 1.** Instruments with performance notes and references for principle of operation and flight-tested implementations. The two mixing ratios for HDO correspond to the range associated with the type of perturbative experiment under consideration, and with its naturally occurring abundance.

instrument	notes	references
aerosol generator	1 kg of liquid H_2_SO_4_ is sufficient to create approximately 3.0×10^7^ m^3^ (100 m radius by 2 km length cylinder) of 15 μm^2^ cm^−3^ surface area density	
H_2_O/HDO injector	10 kg of liquid H_2_O/HDO is sufficient to generate 10 ppmv enhancement over approximately 3.0×10^7^ m^3^	
aerosol counter	1054 nm scatterometer with 100 size bins can measure 0.06–1 μm particles, 3000 particles s^−1^	[73,74]
H_2_O	direct absorption in infrared with Herriot cell: 5% ± 0.2 ppmv accuracy; 2% precision in 1 s	[75]
HDO	mid-infrared integrated cavity output spectroscopy; SNR approximately 10^5^ in 1 s at 10 ppmv, SNR approximately 5 at 1 s and 0.5 ppbv	[76,77]
LIDAR	532 nm micropulse Light Detection and Ranging (LIDAR), integrated to scan mechanism and mounted with clear view for hemispheric scan; range resolution 30/75 m, integration time 1 s	[78,79]
NO_2_	mid-infrared integrated cavity output spectroscopy; SNR approximately 40 in 1 s at 1 ppbv	[77,80–82]
HCl	mid-infrared integrated cavity output spectroscopy; SNR approximately 40 in 1 s at 1 ppbv	[77,80–82]
BrO	chemical conversion–atomic resonance scattering technique with flight-tested inlet design; SNR approximately 10 at 1 s and 10 pptv	[83,84]
ClO	chemical conversion–atomic resonance scattering technique with flight-tested inlet design; SNR approximately 10 at 1 s and 10 pptv	[83,85–87]
O_3_	accuracy 2% or better, precision 2% in 10 s	

Independent variable perturbation (the perturbations to aerosol SAD and water vapour created by SCoPEx constitute the independent variables in the experimental analysis plan):

— aerosol injection: a vapourizer and storage tank provide the material and means to create sulfate aerosol particles of appropriate size;
— H_2_O/HDO vapour injection: a combination of vapourizer and storage tank allow the elevation of the water vapour level.

Independent variable measurement:

— aerosol sizing counter: this measurement counts the number of aerosol particles within size bins to track microphysical evolution, constrain heterogeneous reactivity and allow computation of radiative forcing;
— H_2_O/HDO: H_2_O concentration is a fundamental determinant of reaction rates, and HDO provides a convenient means of distinguishing perturbed from background air; and
— LIDAR: LIDAR plus scanning mechanism to monitor the location of the aerosol plume relative to the balloon platform.

Dependent variable measurement (these measurements detect the response in atmospheric composition to the SAD and water vapour perturbations):

— HCl: direct measurement of HCl quantifies the removal of inert inorganic Cl from its dominant reservoir.
— NO_2_: as discussed in §1*a*, changes in the mixing ratio of photochemically linked NO_2_ or NO are related to the potential for halogen activation.
— BrO: direct measurement of the BrO radical will be performed to constrain ozone loss rates.
— ClO: direct measurement of the ClO radical will be performed to quantify chlorine activation.
— Ozone: *in situ* ozone measurement during perturbation experiments can reveal deviations in ozone loss rate from expectations based on existing photochemical data.

## 3. System architecture

We are following a phased approach to experiment development to reduce project risk, manage costs and to allow disciplined modifications to mission design. To date, we have studied several system architectures for SCoPEx, drawing on a suite of engineering studies, some specific to SCoPEx and others developed for other stratospheric science missions.

The general architecture of such a system consists of a scientific balloon suspending a propeller-driven module that also serves as the injection device for introducing commanded combinations of sulfate aerosol and water as defined above. The distance between the balloon and the suspended module can be adjusted such that the perturbed volume may be tracked and repeatedly sampled with *in situ* instrumentation. The system must allow continuous position surveillance of the perturbed region and repeated opportunities to transit the aerosol and chemical sensors between the perturbed air mass and background air.

Here, we present two plausible specific system architectures denoted as stage one and two. Prior to a decision that would commit funds to building flight hardware, we plan to do further engineering to refine these architectures in a succeeding study that corresponds to phase A in the NASA *Systems engineering handbook* [88]. The resulting mission design might adapt a staged development approach that moved from stages one to two as defined here, or it might proceed directly to a hybrid system.

Both architectures share a set of common design elements, including

— utilization of scientific balloons, either overpressured zero pressure (OZP) [89] or SPB designs;
— altitude control using a winch building on heritage from the ‘reeldown’ system [90,91] and flown in the stratosphere with a tested extension length of 13 km. Although for SCoPEX, an extension length no longer than approximately 1 km is required; and
— propulsion systems that have been deployed for stratospheric airships [92] and have been flight tested for numerous robotic aircraft [93] developed for high-altitude observations. The requirements here are well within the envelope of previous flight systems.

### (a) Stage one system architecture

This stage comprises a single integrated balloon-suspended gondola that includes

— an OZP balloon at a float altitude of approximately 20 km with a system operating endurance of more than 36 h;
— a combination of water ballast dumping and vent controls that allows altitude control to ±0.5 km over a diurnal cycle;
— a winching system capable of maximum extensions of 1 km and vertical rates of 1 km h^−1^ sufficient to maintain altitude control in the face of the balloon's intrinsic altitude variability; and
— finally, a propulsion module capable of driving the system at relative air speeds of up to 1 m s^−1^.

### (b) Stage one concept of operations

This system depends on launching during the low winds found at stratospheric ‘turn around’ [94] as the drive velocity is not sufficient for station keeping during the higher winds prevalent at other times of year. Operational constraints would therefore be similar to that of unpropelled balloons used for stratospheric science and astronomy. This means there is a significant chance that one would not get acceptable conditions during a given season and would miss a launch opportunity.

The balloon launch will be timed so that a plume can be created before dawn. After achieving a stable float altitude chosen to avoid regions of shear-induced turbulence, the perturbed volume would be created, following the same approach for either stage one or stage two architecture, as follows. The perturbing material will be injected into the propeller wake for approximately 1000 s, creating a plume roughly 1 km long with an initial maximum radius of approximately 20 m (figure 2). Plume growth then slows dramatically as propeller wake energy is dissipated: in the absence of vigorous mixing by stratospheric turbulence, the radius remains of order 100 m yielding a total volume of approximately 0.03 km^3^ over the experiment duration.

The payload will then be manoeuvred to fly back and forth through the plume for the duration of the experiment. Operational control of the payload will depend primarily on imaging of the plume using scanning LIDAR which has very high SNR for our particle density at a range of less than 10 km. To assist operational decisions, the payload position orientation (from GPS) will be integrated with LIDAR data to provide the operators with a plume density map referenced to a fixed orientation and the mean drift velocity. Even in cases where experiments do not call for aerosol perturbations, several ‘puffs’ of aerosols will be injected over the 1 km plume length that will provide LIDAR returns for tracking the plume location and shape. If initial experiments show that this is insufficient for navigation, we will supplement knowledge of the plume's location by one or two constant altitude floats with GPS relays [95].

Data from science sensors (e.g. aerosols, H_2_O, HCl, NO_2_, ClO, BrO and O_3_) and analysis by the science team may be used to confirm flight through the plume and to adjust flight profiles. The baseline flight profile would re-enter the plume at multiple points along its length to avoid contamination of plume chemistry by outgassing from the payload.

A central uncertainty in planning operations is the difficulty of predicting plume behaviour under realistic wind shear and turbulence conditions. Early flights will focus on quantitative validation of plume dynamics and on developing the ability to re-enter the plume in a controlled manner.

An advantage of this system architecture is that it does not require an expensive (US$500 000) SPB. It enables engineering tests for initial deployment and system-level integration of the particle generation, LIDAR, propulsion, chemical measurements and winch. However, it is possible that the planned airspeed of 1 m s^−1^ may be insufficient to generate wall-less intake flows for the ClO and BrO sensors.

### (c) Stage two system architecture

The stage two architecture is derived from engineering work performed in support of the Airborne Stratospheric Climate Coupled Convective Catalytic Chemistry Experiment North America (ASC^5^ENA) mission proposal [96], which is designed to test hypotheses about stratospheric chemistry, dynamics and mid-latitude convection. This mission proposal has been submitted to NASA as an Earth Venture Suborbital investigation and engineering work is currently supported by two SBIR grants [80,97].

**Figure 3.**
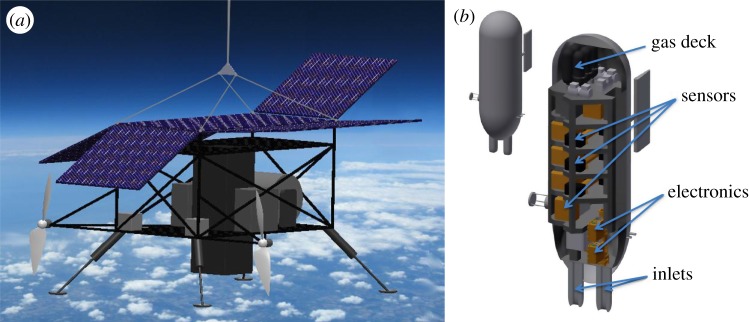
The StratoCruiser propulsion module (*a*) contains the docking enclosure for the suspended payload, the articulated solar panels for power, Li–Po batteries for energy storage, dual high-efficiency propellers for concerted directional control, the winching system for suspended payload reeldown as well as all electronics support and command/control requirements. A cutaway of the suspended payload (*b*) shows representative *in situ* instruments and their associated inlet systems, meteorological measurements, electronics support, communication command and control, and safety parachute. The configuration of sensors for SCoPEx will be finalized in future engineering studies.

The ASC^5^ENA system consists of a superpressure ‘pumpkin’ [55] balloon suspending a drive unit, designated the ‘StratoCruiser’ propulsion module, that itself suspends a separate winch-driven sensor payload (figure 3). This StratoCruiser system significantly augments the capabilities of the SCoPEx stage one experimental system, including but not limited to:

— the capability to drive at up to 8 m s^−1^ relative to background winds;
— articulated solar panels to fully provide the power necessary to drive the system and perform the science functions;
— the capability to perform vertical soundings of up to 10 km using the ‘reeldown’ winching system, controlling the vertical position of the suspended payload at controlled rates of up to 10 m s^−1^;
— an augmented sensor array, including atmospheric tracer species CO_2_, CO, N_2_O and CH_4_, enhanced wind measurements, two digital cameras and measurement of condensed phase water and the HDO isotopologue; and
— the combination of the superior drive capability and solar panels allows an augmentation of the experimental lifetime up to six weeks.

The StratoCruiser system can be modified to implement the SCoPEx perturbative experimental concept, leading to a system we designate as the SCoPEx stage two. The propulsion module can be engineered to accommodate a sulfate–water injection system and a winching system to adjust its distance to the balloon.

### (d) Stage two concept of operations

**Figure 4.**
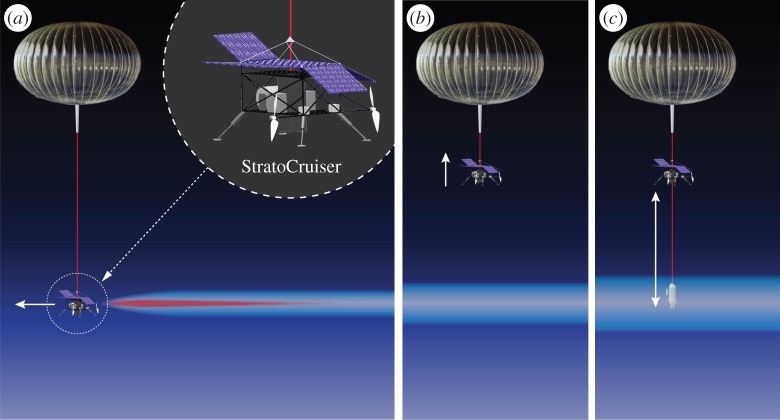
The concept of operations for the proposed experiment is initiated by seeding a 1 km length of stratospheric air with a combination of water vapour and sulfate aerosol using the propulsive capability of the StratoCruiser (*a*). Using a combination of its altitude and propulsive capabilities, the StratoCruiser manoeuvres past and above the seeded volume, which continues to expand owing to the turbulent wake generated by the propellers. The suspended instrument payload is reeled through the seeded volume to measure aerosols, water vapour and chemical species including HCl and ClO (*b*). The propulsion capability together with the LIDAR surveillance is used to track the seeded volume as it drifts with ambient wind and to make repeated measurements with the suspended payload, resolving the chemical evolution within the seeded volume as a function of time (*c*).

The concept of operations for stage two would proceed in a conceptually similar way to stage one. In stage two, the system will be launched and allowed to achieve float altitude. Because of its extended lifetime, the system will be allowed to dwell at float altitude for a pre-operational period, during which it observes the local meteorology. Based on these meteorological observations, the science team will select an air mass for experimentation based on its temperature and wind shear. The StratoCruiser propulsion module will then inject commanded combinations of water and sulfate as defined in stage one, leading to a well-mixed perturbed plume approximately 1 km in length and order 100 m in radius. The distance between the balloon and the StratoCuiser can be adjusted over a vertical range of 1 km such that the propulsion module can perturb the desired volume (which has been tested for quiescent conditions) and then retract to a position approximately 1 km above the seeded region, tracking the volume with LIDAR to maintain continuous position surveillance of the measurement region and remain directly over the seeded volume (figure 4). The suspended payload that contains the array of *in situ* instruments can then be lowered into the seeded region multiple times. This experimental protocol is consistent with a set of operating procedures developed in partnership with the Columbia Scientific Balloon Facility for ASC^5^ENA that permit safe operation within a large designated airspace for a mission lasting six weeks during the months of June–August [96].

The enhanced capabilities of the stage two StratoCruiser system over stage one architecture substantially reduce the risk of failing to obtain a viable experimental operating window and increase the scientific returns, including but not limited to:

— the augmented drive capability allows safe operation during times of year of higher stratospheric winds beyond the short turnaround periods in late spring and early autumn. By expanding the operational window to include June–August, the probability of gaining a launch window and completing a successful experiment campaign is markedly improved;
— the experimental system can, on a single flight, run the injection and sampling protocol multiple times;
— the controlled descent rate of the suspended payload ensures the isolation radical molecules in the inlet air stream from the walls of the ClO and BrO sensors;
— the system has greater latitude to select from a range of background meteorological conditions, adding a further degree of control to the experimental protocol; and
— the measurement of tracer species CO_2_, CO, N_2_O and CH_4_ ties all measurements to a widely used set of chemical coordinates [98,99], facilitating comparability with other stratospheric chemistry observations that include similar tracers, regardless of measurement platform (aircraft, balloon, satellite).

## 4. Expected results and data analysis

### (a) Perturbation and anticipated response

SCoPEx will perform a suite of experiments to improve our understanding of aerosol microphysics and heterogeneous ozone chemistry. We have formulated a baseline experiment to allow quantitative evaluation of the experimental design via engineering analysis and chemical modelling.

The preliminary experimental range is defined by

— background atmospheric conditions:
  temperatures: 200–210 K, 5 ppmv H_2_O, 2 μm^2^ cm^−3^ aerosol SAD;
— plume nominal volume: 0.03 km^3^, radius of order 100 m by 1 km long;
— plume perturbations:
  range of sulfate aerosol SAD increases of 10–50 μm^2^ cm^−3^
  range of water vapour increases of 5–15 ppmv, to totals of 10–20 ppmv.

**Figure 5.**
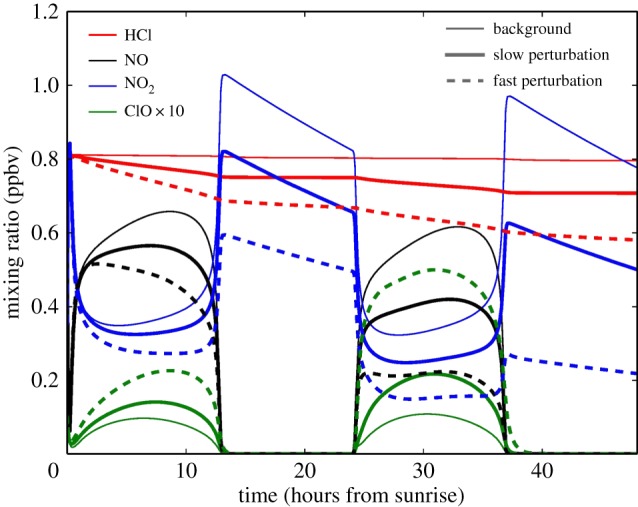
Calculated concentrations (ppbv) of HCl, NO, NO_2_ and ClO under background conditions (thin solid lines), and ‘slow’ (solid thick lines) and ‘fast’ (dashed thick lines) perturbed conditions for 48 h following injection that occurs just before dawn. See details in §4*a*. Note that ClO concentrations have been scaled up by a factor of 10 for clarity. The background conditions are 5 ppmv H_2_O and 2 μm^2^ cm^−3^ SAD sulfate aerosol. ‘Slow’ case has *T*=208 K and 15 SAD μm^2^ cm^−3^; ‘fast’ case has *T*=204 K and 50 μm^2^ cm^−3^ SAD. Both cases have 10 ppmv H_2_O inside plume.

To provide confidence that the chemical perturbations that would be generated in the SCoPEx experiment can be detected by the proposed instruments, we have performed simulation of the chemical dynamics. We use a box model that is equivalent to a single grid cell of the AER two-dimensional model [100] situated at 38°N and 64 hPa in September. Chemical reaction rates are from Sander *et al.* [101], and calculations are initialized with results from the free-running global two-dimensional model at this location and date. While the plume would continue to expand over the 48 h of the experiment, these calculations assume a constant H_2_O mixing ratio and sulfate aerosol SAD inside the plume. We consider two limiting cases: a ‘slow’ perturbation with aerosol SAD of 15 μm^2^ cm^−3^, H_2_O of 10 ppm and a temperature of 208 K, and a ‘fast’ perturbation with aerosol SAD of 50 μm^2^ cm^−3^, H_2_O mixing ratio of 10 ppm and a temperature of 204 K. We compute the evolution of chemical constituents inside and outside the plume. Figure 5 shows the concentrations of HCl, NO, NO_2_ and ClO during 48 h following an injection of H_2_O and H_2_SO_4_ that occurs just before dawn. The ‘slow’ case implies a decrease of HCl of only 8% over the first 12 h, providing a sensitive test of the capability of the perturbative experiment approach to disentangle small induced changes in composition from fluctuations owing to natural variability. The ‘fast’ case demonstrates the increase in photochemical reaction rates that occurs when colder temperatures and higher SAD combine to double the decrease in HCl that occurs in the first 12 h.

Quantitative analysis of reaction rates from observations will be greatly aided by the use of HDO to label the perturbed air. We will, for example, plot the ratio of the HCl to HDO/H_2_O where the HDO/H_2_O ratio will serve as a very high SNR tracer of plume dilution. While changes in HCl may be hard to detect, even in the ‘slow’ case, ClO shows a 45% increase in the first 8 hours, and an increase of approximately 100% in the second diurnal cycle.

## 5. Governance

SRM experiments are controversial—and rightly so, for SRM carries substantial risks, and there are legitimate arguments against this research. The direct environmental risks of SCoPEx are very small: less than 1 kg of sulfuric acid is needed per flight, an amount that is less than the amount of sulfur released by one commercial passenger jet in 1 min of flight time (table 2). Whatever the physical risks are, the SCoPEx mission is committed to fostering a fully independent risk assessment and approval process using mechanisms such as an environmental assessment under the National Environmental Policy Act.

**Table 2.** Comparison of perturbations between SCoPEx and commercial air transport.

source	H_2_O (kg)	H_2_SO_4_ (kg)
commercial jet		
1 transatlantic flight of 5000 km, 6 h	140 000	180
SCoPEx		
baseline plume of 1 km	5	0.5

Quite distinct from the physical risks, there are other concerns about geoengineering research that arise from the potential for socio-technical lock-in [75]. While a thorough review of this topic is beyond the scope of this paper, SCoPEx has some distinctive features shaping its potential risks. While it is possible to perturb the lower stratosphere with SCoPEx for the purposes of testing key aspects of SRM, the cost of scaling SCoPEx as a deployment method is so prohibitive that the development of the SCoPEx experiment would not directly accelerate the development of hardware, industrial infrastructure or operational methods relevant to deployment. Whatever our judgement of these risks, we will only proceed with SCoPEx if it passes independent risk assessment and if it is financed predominantly with public funding from a relevant scientific agency.

### (a) Safety

Management of safety issues associated with SCoPEx (table 3) is of primary importance. These issues are associated with the operation of scientific equipment, with scientific ballooning and with chemical perturbations created by aerosol particles or water vapour additions. Maintaining safe deployment of the planned chemical perturbations will be achieved in accordance with recommendations provided through external oversight. Our current scientific and operational planning suggest that the science objectives can be achieved with total perturbation less than 1 kg H_2_SO_4_ and less than 10 kg H_2_O. These perturbations are small compared with common aircraft activities. For example, a commercial aircraft emits roughly equal amounts of sulfur and water in less than 2 min of flight time, and such aircraft do routinely fly in the stratosphere.

**Table 3.** Risks and mitigation.

	risk	description/assessment	mitigation
	risks to operators	concentrated sulfuric acid, high-power lasers, high-pressure gas cylinders, propeller	standard safety procedures for caustics, lasers, gas cylinders; propeller fixed during launch phase
	risks to public on the ground	debris, uncontrolled recovery	operation in area of low population density, standard flight safety procedures
	risks to aircraft	air traffic concerns	FAA beacon and coordination operational altitude >65 000 feet
	risks from H_2_O release	chemical/radiative perturbation to stratosphere	none anticipated to be necessary
	risks from H_2_SO_4_ release	chemical/radiative perturbation to stratosphere	none anticipated to be necessary

## 6. Summary

The development of stratospheric airships, SPBs and propulsion systems over more than three decades provides the engineering foundation for rapid, low-risk development of the SCoPEx platform. Our choice of a novel propelled balloon platform stems from the limited ability of existing stratospheric aircraft or balloons to meet the mission science requirements of low-velocity and long duration during periods of very light winds and low shear that occur on a seasonal basis in the lower stratosphere.

The scientific instruments build directly on a decades-long history of stratospheric composition measurements [76,77,81–87,102,103]. These instruments provide high temporal resolution and high sensitivity to allow sampling of subtle chemical gradients that can be used to infer the time dependence of chemical reactions. These small-scale features cannot be measured by remote sensing methods that average over large spatial footprints, erasing essential information about chemical reactivity. The measurements made by SCoPEx provide context for measurements made on larger spatial scales and at longer time scales, bridging the gap between small-scale processes and prediction of the atmosphere's response to large-scale forcing.

To be clear, while the small-scale nature of SCoPEx minimizes a number of risks, it also leaves a number of key uncertainties for other investigations. These include potential variations in aerosol microphysics arising from varying meteorological conditions, different aircraft wake characteristics and other particle generation techniques. There are also numerous uncertainties associated with geoengineering deployment—changes to large-scale atmospheric circulations and aerosol deposition at the surface [104], to name two—that are not addressed by SCoPEx.

External oversight and adherence to established safety practices are an essential part of the SCoPEx approach to risk management. The physical risks associated with scientific ballooning and custom instrumentation are managed using standard methods applied across all balloon missions. The size of the chemical perturbations in SCoPEx is tiny relative to chemical perturbations caused by a few minutes of flight of a commercial passenger aircraft.

In summary, we have presented a case for an outdoor experiment to test the risks and efficacy of SRM. The motivation for outdoor experimentation is grounded in a larger scientific context and in the need to reduce uncertainties inherent in representing the complex atmospheric system in the laboratory, by a natural analogue, or in a model. The scientific results are expected to inform theoretical predictions about stratospheric composition in a changing climate with high-resolution, high-accuracy data.
